# Outstanding Reviewers for *Chemical Science* in 2023

**DOI:** 10.1039/d4sc90147a

**Published:** 2024-08-02

## Abstract

We would like to take this opportunity to thank all of *Chemical Science*'s reviewers for helping to preserve quality and integrity in chemical science literature. We would also like to highlight the Outstanding Reviewers for *Chemical Science* in 2023.

## Abstract 2

We would like to take this opportunity to highlight the Outstanding Reviewers for *Chemical Science* in 2023, as selected by the editorial team for their significant contribution to the journal.

## Graphical abstract

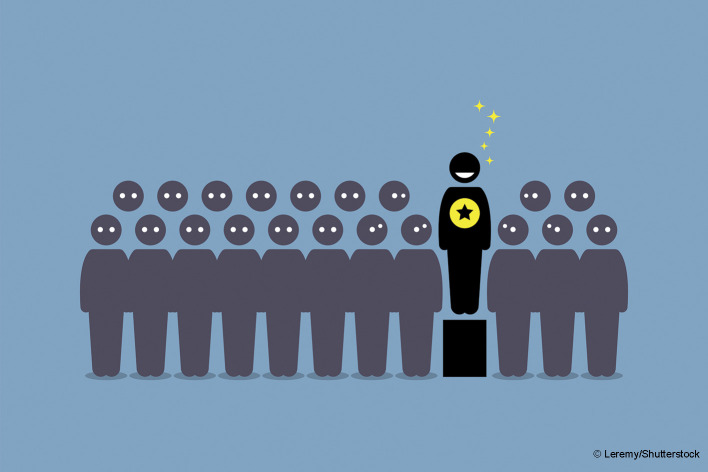

Each one of our outstanding peer reviewers has been carefully selected by our editorial team and includes active researchers who have made significant contributions to peer review and have gone above and beyond in their actions.

As well as selecting reviewers who have provided a higher-than-average number of top-quality reviewer reports, we have also chosen to highlight those reviewers who have provided highly detailed reports and provided constructive feedback, as well as those adjudicative reviewers who have provided thoughtful and robust reports for manuscripts that have initially received conflicting recommendations.

In addition to our list of Outstanding Reviewers for *Chemical Science*, we are highlighting our superb reviewer community year-round in our Reviewer Spotlight blog, which highlights a selection of reviewers every month. You can read our Reviewer Spotlight blog at https://blogs.rsc.org/sc/category/reviewer-spotlight/ and do keep an eye on our journal Twitter/X account @ChemicalScience to see which reviewers we will be promoting each month.

 

Dr Lutz Ackermann

University of Göttingen

ORCID: 0000-0001-7034-8772

 

Dr Sarit Agasti

Jawaharlal Nehru Centre for Advanced Scientific Research (JNCASR)

ORCID: 0000-0001-7216-0490

 

Dr Ulf-Peter Apfel

Ruhr-Universität Bochum

ORCID: 0000-0002-1577-2420

 

Dr Claudia Andrea Blindauer

University of Warwick

ORCID: 0000-0001-8396-9332

 

Professor Katerine Bujold

McMaster University

ORCID: 0000-0002-7292-6617

 

Dr Scott Lee Cockroft

University of Edinburgh

ORCID: 0000-0001-9321-8997

 

Professor Sabrina Conoci

University of Messina

ORCID: 0000-0002-5874-7284

 

Dr Owen Curnow

University of Canterbury

ORCID: 0000-0001-7479-856X

 

Dr Sascha Feldmann

Harvard University (USA) & EPFL (Switzerland)

ORCID: 0000-0002-6583-5354

 

Dr Andrea Fermi

University of Bologna

ORCID: 0000-0003-1080-0530

 

Dr Jennifer Anne Garden

University of Edinburgh

ORCID: 0000-0002-9027-4931

 

Dr Mariateresa Giustiniano

University of Naples Federico II

ORCID: 0000-0002-6856-414X

 

Dr Malcolm Halcrow

University of Leeds

ORCID: 0000-0001-7491-9034

 

Professor Mahesh Hariharan

Indian Institute of Science Education and Research Thiruvananthapuram

ORCID: 0000-0002-3237-6235

 

Dr Fiona Hatton

Loughborough University

ORCID: 0000-0002-0105-7530

 

Dr Chuan He

Southern University of Science and Technology

ORCID: 0000-0002-9983-7526

 

Dr Esther Heid

Technische Universität Wien

ORCID: 0000-0002-8404-6596

 

Dr Laura M. Hernandez

McGill University

ORCID: 0000-0002-1078-8597

 

Professor Shuichi Hiraoka

University of Tokyo

ORCID: 0000-0002-9262-4747

 

Dr Hiroshi Imahori

Kyoto University

ORCID: 0000-0003-3506-5608

 

Dr Kazuaki Ishihara

Nagoya University

ORCID: 0000-0003-4191-3845

 

Dr Ranjan Jana

CSIR-Indian Institute of Chemical Biology

ORCID: 0000-0002-5473-0258

 

Dr Niveen M. Khahsab

King Abdullah University of Science and Technology

ORCID: 0000-0003-2728-0666

 

Dr Isabelle Landrieu

CNRS Integrative Structural Biology team (EMR 9002), Lille France

ORCID: 0000-0002-4883-2637

 

Dr Bettina V. Lotsch

Max Planck Institute for Solid State Research

ORCID: 0000-0002-3094-303X

 

Professor Klaus Braagaard Møller

Technical University of Denmark

ORCID: 0000-0002-9797-7437

 

Dr John Murphy

University of Strathclyde

ORCID: 0000-0003-3136-0845

 

Dr Angshuman Nag

Indian Institute of Science Education and Research Pune

ORCID: 0000-0003-2308-334X

 

Dr Manuel Nappi

CiQUS – Universidade de Santiago de Compostela

ORCID: 0000-0002-3023-0574

 

Professor Sharon R. Neufeldt

Montana State University

ORCID: 0000-0001-7995-3995

 

Dr Timothy Noël

University of Amsterdam

ORCID: 0000-0002-3107-6927

 

Dr Marina Petrukhina

University at Albany – State University of New York

ORCID: 0000-0003-0221-7900

 

Dr Sarah Pike

University of Birmingham

ORCID: 0000-0002-1235-0265

 

Dr Elisabeth Prince

University of Waterloo

ORCID: 0000-0003-2948-4603

 

Dr Kanyi Pu

Nanyang Technological University

ORCID: 0000-0002-8064-6009

 

Dr Davide Ravelli

University of Pavia

ORCID: 0000-0003-2201-4828

 

Dr Patricia Rodriguez Macia

University of Leicester

ORCID: 0000-0003-3513-0115

 

Dr Mattia Silvi

University of Nottingham

ORCID: 0000-0002-0728-7193

 

Dr Nicholas White

Australian National University

ORCID: 0000-0003-2975-0887

 

Professor Haoxing Wu

Sichuan University

ORCID: 0000-0002-0008-4668

 

Dr Ming Xian

Brown University

ORCID: 0000-0002-7902-2987

 

Professor Junpei Yuasa

Tokyo University of Science

ORCID: 0000-0003-1117-7904

 

Professor Tierui Zhang

Technical Institute of Physics and Chemistry, Chinese Academy of Sciences

ORCID: 0000-0002-7948-9413

 

Dr Jenny Z. Zhang

University of Cambridge

ORCID: 0000-0003-4407-5621

 

Dr Xiyue Zhang

University of Maryland

ORCID: 0000-0002-3462-1548

 

We would also like to thank the *Chemical Science* Editorial Board and Advisory Board and the chemical sciences community for their continued support of the journal, as authors, reviewers and readers.

We continue to work on improving the diversity of our reviewer pool to reflect the diversity of the communities that we serve.

 

May Copsey, Executive Editor

